# Quantification of ischemic brain edema after mechanical thrombectomy using dual-energy computed tomography in patients with ischemic stroke

**DOI:** 10.1038/s41598-024-54600-0

**Published:** 2024-02-20

**Authors:** Paul Steffen, Laurens Winkelmeier, Helge Kniep, Vincent Geest, Setareh Soltanipanah, Jens Fiehler, Gabriel Broocks

**Affiliations:** Department of Diagnostic and Interventional Neuroradiology, University Medical Center Hamburg-Eppendorf, Martinistraße 52, 20251 Hamburg, Germany

**Keywords:** Neurology, Outcomes research

## Abstract

Net water uptake (NWU) is a quantitative imaging biomarker used to assess cerebral edema resulting from ischemia via Computed Tomography (CT)-densitometry. It serves as a strong predictor of clinical outcome. Nevertheless, NWU measurements on follow-up CT scans after mechanical thrombectomy (MT) can be affected by contrast staining. To improve the accuracy of edema estimation, virtual non-contrast images (VNC-I) from dual-energy CT scans (DECT) were compared to conventional polychromatic CT images (CP-I) in this study. We examined NWU measurements derived from VNC-I and CP-I to assess their agreement and predictive value in clinical outcome. 88 consecutive patients who received DECT as follow-up after MT were included. NWU was quantified on CP-I (cNWU) and VNC-I (vNWU). The clinical endpoint was functional independence at discharge. cNWU and vNWU were highly correlated (r = 0.71, *p* < 0.0001). The median difference between cNWU and vNWU was 8.7% (IQR: 4.5–14.1%), associated with successful vessel recanalization (mTICI2b-3) (ß: 11.6%, 95% CI 2.9–23.0%, *p* = 0.04), and age (ß: 4.2%, 95% CI 1.3–7.0%, *p* = 0.005). The diagnostic accuracy to classify outcome between cNWU and vNWU was similar (AUC:0.78 versus 0.77). Although there was an 8.7% median difference, indicating potential edema underestimation on CP-I, it did not have short-term clinical implications.

## Introduction

Randomized control trials have established endovascular mechanical thrombectomy (MT) for acute ischemic stroke as the standard of care for patients with acute large vessel occlusion (LVO)^1^. The degree of recanalization on the modified Thrombolysis in Cerebral Infarction (mTICI) scale correlates with functional outcome^2,3^ and a score of mTIC2c or 3 should be strived for^4^. Yet, the functional outcome even with same mTICI scores varies widely^5^ and is influenced by different parameters such as clinical onset to successful reperfusion time, patient age, the National Institutes of Health Stroke Scale (NIHSS) at admission, the Alberta stroke program early computed tomography (ASPECT) score at admission, and the baseline functional status^6^. Furthermore, tissue infarction and ischemic edema are two compartments of ischemic lesions and independent predictors of clinical outcome^7,8^.

Ischemic edema after LVO can be measured using net water uptake (NWU) in early follow-up computed tomography (CT). NWU is a quantitative imaging biomarker and indicator of the response to MT^9^, which showed to be of high value as a tool for outcome prediction complementary to other clinical variables such as age, National Institutes of Health Stroke Scale (NIHSS), and Alberta Stroke Program Early CT Score (ASPECTS)^9^. Other studies also described a strong correlation between NWU measured in follow-up CT and final infarct volume^7,10^.

Ischemic edema is the pathophysiological response of ischemic brain tissue undergoing infarction. Water influx in the infarcted brain area leads to progressive tissue hypoattenuation^11^. Disruption of the blood–brain-barrier due to ischemia may not only lead to edema but also to hemorrhage and contrast staining after MT^12^. Hyperattenuating lesions are common findings after MT for LVO^13^, and pose a difficulty for NWU quantification on CP-I. In line with this, a recent pilot study observed that in 20% of the patients, edema was significantly underestimated when using densitometry in CP-I^14^.

Research on dual-energy CT (DECT) imaging has led to practical applications due to its capability to distinguish between tissues of similar x-ray attenuation but different atomic numbers^15–19^. This discrimination is possible due to specific absorption characteristics of different materials because of photoelectric and Compton scattering. Since both phenomena depend on the applied x-ray photon energy, scanning at two different energy levels allow to discriminate the pixel attenuation arising from these two effects^18^. Iodine for instance increases x-ray absorption at 32.2 keV^16,20^. These specific absorption characteristics are captured and used to generate dual-energy CT datasets which can be used to calculate virtual non-contrast images (VNC-I)^14^.

Differentiation between tissues of similar Hounsfield Units, such as hemorrhage and contrast staining, remains difficult on CP-I^21^. VNC-I however, shows promising results to correctly identify hyperattenuating lesions after LVO as intracranial hemorrhage with high sensitivity^18,22^, and therefore may improve NWU quantification and outcome prediction^14^.

Our study aimed to assess the potential impact of contrast staining on conventional follow-up CT scans after MT, which could lead to underestimation of edema and hinder the predictive accuracy of NWU quantification for clinical outcome. Furthermore, we explored the potential benefits of utilizing VNC-I instead of CP-I for NWU calculation to improve accuracy.

## Materials and methods

### Study design and study population

The local ethics committee (of University Medical Hospital Hamburg-Eppendorf, Hamburg; Number WF 04/13) approved the study and waived the requirement to obtain informed consent. The design and procedures of the study were carried out in accordance with the principles of the Declaration of Helsinki. This retrospective single-center study investigated whether dual-energy CT scans using VNC-I are superior for the assessment of NWU in acute stroke patients for clinical outcome prediction compared to traditional polychromatic CT scans. The local ethics committee approved the study and waived the requirement to obtain informed consent. We anonymized and retrospectively analyzed the data of 88 consecutive patients referred to our hospital between June 2020 and December 2021 with LVO of the anterior circulation who received MT. Inclusion criteria were defined as follows: (1) acute ischemic stroke in the anterior circulation due to an isolated occlusion of the intracranial carotid bifurcation or of the M1 or M2 segment of the middle cerebral artery confirmed on admission CT; (2) MT was performed according to the ESO/ESMINT-Guidelines^4^; (3) dual-energy follow-up cranial CT scan after MT; (4) visually hypoattenuated and circumscribable infarct lesion on follow-up imaging.

### Image acquisition and reconstruction

All CT scans were performed on a dual-energy dual-layer CT (IQon spectral CT, Philips Healthcare, USA) with the following imaging parameters: nonenhanced CT with 120 kVp, 230 mAs, and 5.0 mm slice reconstruction. As post-processing software IntelliSpace (Version 11.1, Philips Healthcare, USA) was used to generate CP-I and VNC-I from spectral based datasets. The CP-I were reconstructed using iterative model-based reconstructions (level 1, filter UB), whereas the VNC-I were generated using a spectral reconstruction mode (level 2)^14^.

### Image analysis

**Figure 1:**
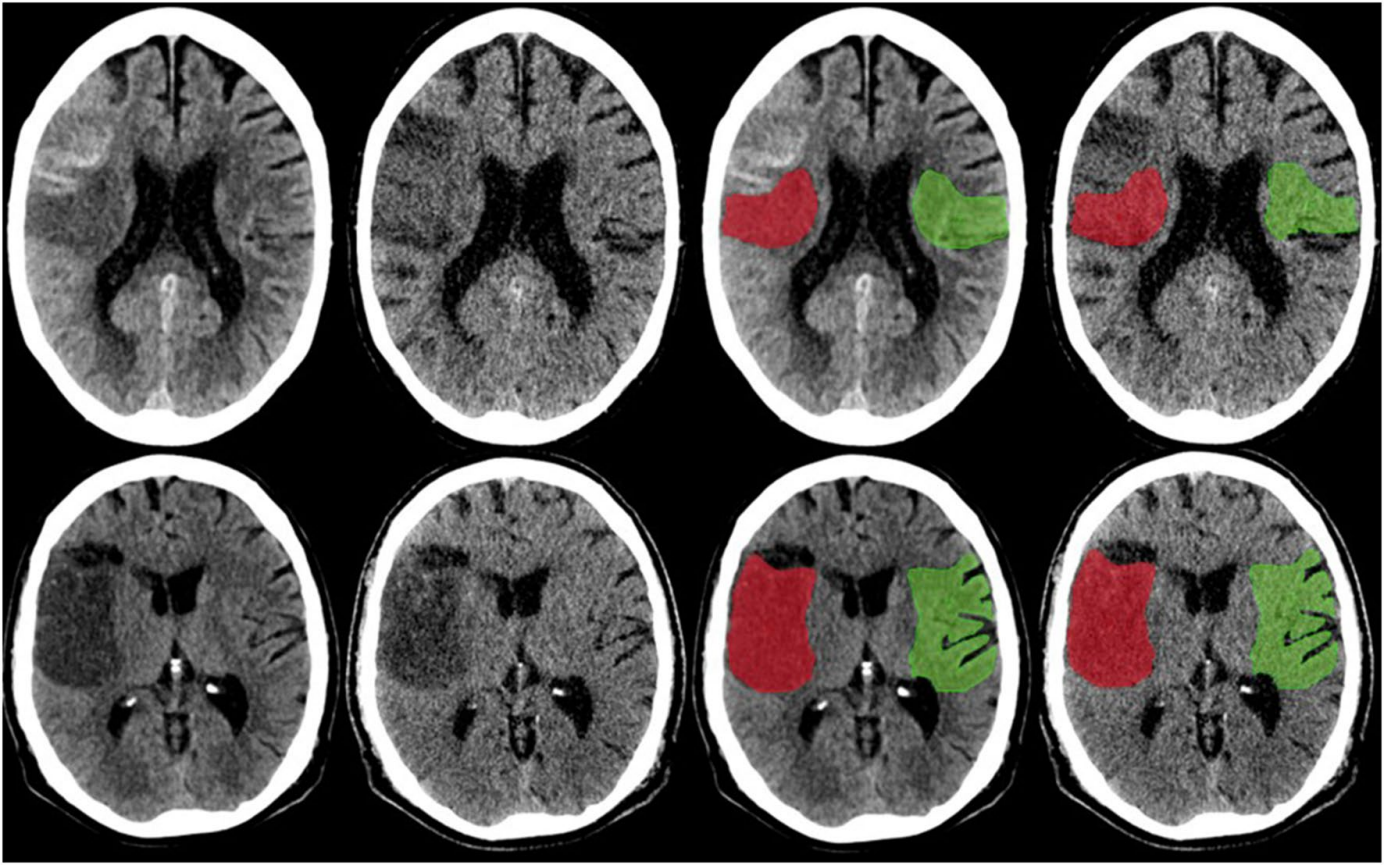
Examples for region of interest (ROI) placements in polychromatic (1st and 3rd images from left) as well as corresponding virtual non-contrast (2nd and 4th images from left) images for net water uptake measurements. The ischemic infarction (Red) was compared to the corresponding unaffected contralateral region (green). Top row: Follow-up dual-energy CT within 16 h in an 84-year-old female with right middle cerebral artery (MCA) occlusion (initial NHISS 20; ASPECTS 7) after thrombectomy (mTICI 0). Example to demonstrate how visually apparent hyperattenuations in conventional polychromatic images (CP-I) were spared during ROI placement. Edema underestimation was 12.8%. Bottom row: Follow-up dual-layer dual-energy CT within 16 h in a 69-year-old man with right MCA occlusion (initial NHISS 17; ASPECTS 4) after thrombectomy (mTICI 2b). Example of ischemic ROI placement if no visually apparent hyperattenuations were seen on CP-I. Edema underestimation was 4.5%.

The data was anonymized and the rater was blinded for all patient related data and clinical information. The rating was done by a neuroradiologist (PS) with > 6 years of experience in stroke image analysis. Results were confirmed by a second neuroradiologist (GB, 7 years of experience). Discrepancies were settled in a consensus reading. The rating was done using a commercially available software (Analyze 11.0, Biomedical Imaging Resource, Mayo Clinic, Rochester, MN). A standardized procedure to quantify the proportion of ischemic edema due to NWU was used as previously published and extended to account for CP-I and VNC-I comparison^7,23^. In summary, ischemic edema was quantified by placing a region of interest (ROI) on a sligle slice on visually evident edematous hypoattenuation on the conventional CT-scans for density measurements with knowledge of the core ischemic lesion from CT perfusion images. A symmetric ROI was mirrored within unaffected brain tissue on the contralateral hemisphere. Sulci were excluded manually. ROI histograms were sampled between 20 and 80 Hounsfield units to exclude voxels belonging to cerebrospinal fluid or calcifications. Both density measurements were used to calculate the proportion of edema (cNWU) as the difference between density of the infarcted area to the unaffected hemisphere. The ROIs from the conventional images were saved and reused for the same person’s VNC-I (for vNWU) to ensure that both, CP-I and VNC-I density measurements were performed at identical regions of the brain. Examples of ROI placement is depicted in Fig. 1. Difference in cNWU and vNWU were calculated to obtain edema underestimation.

### Statistical analysis

The difference between cNWU and vNWU was defined es edema underestimation with higher values indicating an increase in edema underestimation. The median NWU difference between cNWU and vNWU was used to divide the study cohort into two groups of patients (i.e. low edema underestimation versus high edema underestimation). Non-parametrical Kruskal–Wallis/Mann Whitney U-Test was used for continuous variables and were reported as median and interquartile range (IQR). Pearson’s chi-squared test was used for categorical/binary variables and are reported as counts and percentages. cNWU and vNWU were compared using paired sample t-tests with means and 95% confidence intervals. Pairwise Correlation between cNWU and vNWU was tested. Multivariable linear regression analysis was performed with the absolute difference of cNWU and vNWU as dependent variable implementing variables with established impact on edema formation (time from onset, age, ASPECTS, and status of vessel recanalization). Finally, the potential clinical relevance of edema underestimation was tested by comparing two multivariable logistic regression models with mRS 0–2 at discharge as dependent variable. Variables were selected based on established associations to outcome (Age, NIHSS, ASPECTS, and degree of recanalization). For Model 1, cNWU was added, and for Model 2, vNWU was implemented. The area under the curve (AUC) for both models were calculated. Analyses were performed using Stata 17.0 (StataMP, StataCorp, TX, USA), R statistical software (version 4.1.2, R Project for Statistical Computing), and RStudio statistical software (version 2021.09.1 + 372, RStudio). A two-tailed *p* value of < 0.05 was considered statistically significant for all statistical tests.

## Results

### Patient Characteristics and stratification by low and high edema underestimation

88 consecutive patients were assessed for eligibility, of which 70 patients met the inclusion criteria. 18 patients were excluded due to an absence of any infarct demarcation on follow up imaging (14 patients), parenchymal hemorrhage type 2 according to Heidelberg Bleeding Classification^24^ (2), strong artifacts from extracranial metal (1), and reinfarction with thrombectomy before follow-up CT scan (1). Among the included patients, mean Age was 76.1 years (range: 47–93) with 54.3% being female. The median NIHSS score on admission was 13 points (IQR: 8–18) and the baseline ASPECTS was 8 (IQR 7–9). Successful recanalization defined as mTICI 2b, 2c or 3 was achieved in 58 cases (83%) of patients.

Stratified by low (< 8.7%) and high (≥ 8.7%) edema underestimation, no significant differences between groups were observed regarding clinical parameters (NIHSS and mRS) on admission or discharge. No difference on admission ASPECTS was seen. Time between symptom onset to flow restoration differed significantly between the two study groups with 289 min in patients with high edema underestimation compared to 372 min in patients with low edema underestimation (*p* = 0.019). Successful recanalization was achieved in 26 (74.3%) patients with low edema underestimation and in 32 (91.4%) patients with high edema underestimation showing a trend but no significant difference (*p* = 0.057). Table 1 shows baseline, treatment, and outcome characteristics for the two study groups.

**Table 1:** Baseline, treatment, and outcome characteristics stratified by < 8.7% and ≥ 8.7% edema quantification difference.

	Low edema underestimation (N = 35)	High edema underestimation (N = 35)	*p* value
Baseline characteristics			
Female sex, n (%)	15 (42.9)	23 (65.7)	0.055
Age, mean (SD)	73.94 (10.18)	77.31 (10.79)	0.108
HbA1c, mean (SD)	5.97 (0.86)	5.98 (0.43)	0.342
Admission NIHSS, median (IQR)	15 (11.00–17.50)	13 (7.00–17.50)	0.420
Admission mRS, median (IQR)	5 (4–5)	4 (3–5)	0.183
Admission ASPECTS, median (IQR)	8 (7–9)	8 (7–9)	0.976
Treatment characteristics			
Successful recanalisation, n (%)	26 (74.3)	32 (91.4)	0.057
Time admission CT to flow restoration, median (IQR)	119 (95–147)	105 (81–138)	0.123
Time from symptom onset to flow restoration, median (IQR)	372 (335–593)	289 (225–343)	0.019
Time flow restoration to FU CT, median (IQR)	1208 (997–1514)	1263 (985–1439)	0.868
Administration of intravenous thrombolysis, n (%)	16 (45.7)	21 (60.0)	0.231
Outcome characteristics			
Discharge NIHSS, median (IQR)	5.00 (1.00, 16.50)	5.00 (2.00, 14.00)	0.919
Discharge mRS, median (IQR)	3.00 (1.00, 5.00)	3.00 (2.00, 5.00)	0.870

Patient characteristic of subgroups. Successful recanalisation is defined as mTICI 2b or higher. Time data is given in minutes. N number of patients, SD standard deviation, IQR interquartile range, NIHSS National Institutes of Health Stroke Scale, mRS modified Rankin Scale, ASPECTS Alberta Stroke Programme Early CT Score. P-value < 0.05 indicates clinical significance.

### Correlation between cNWU and vNWU

**Figure 2:**
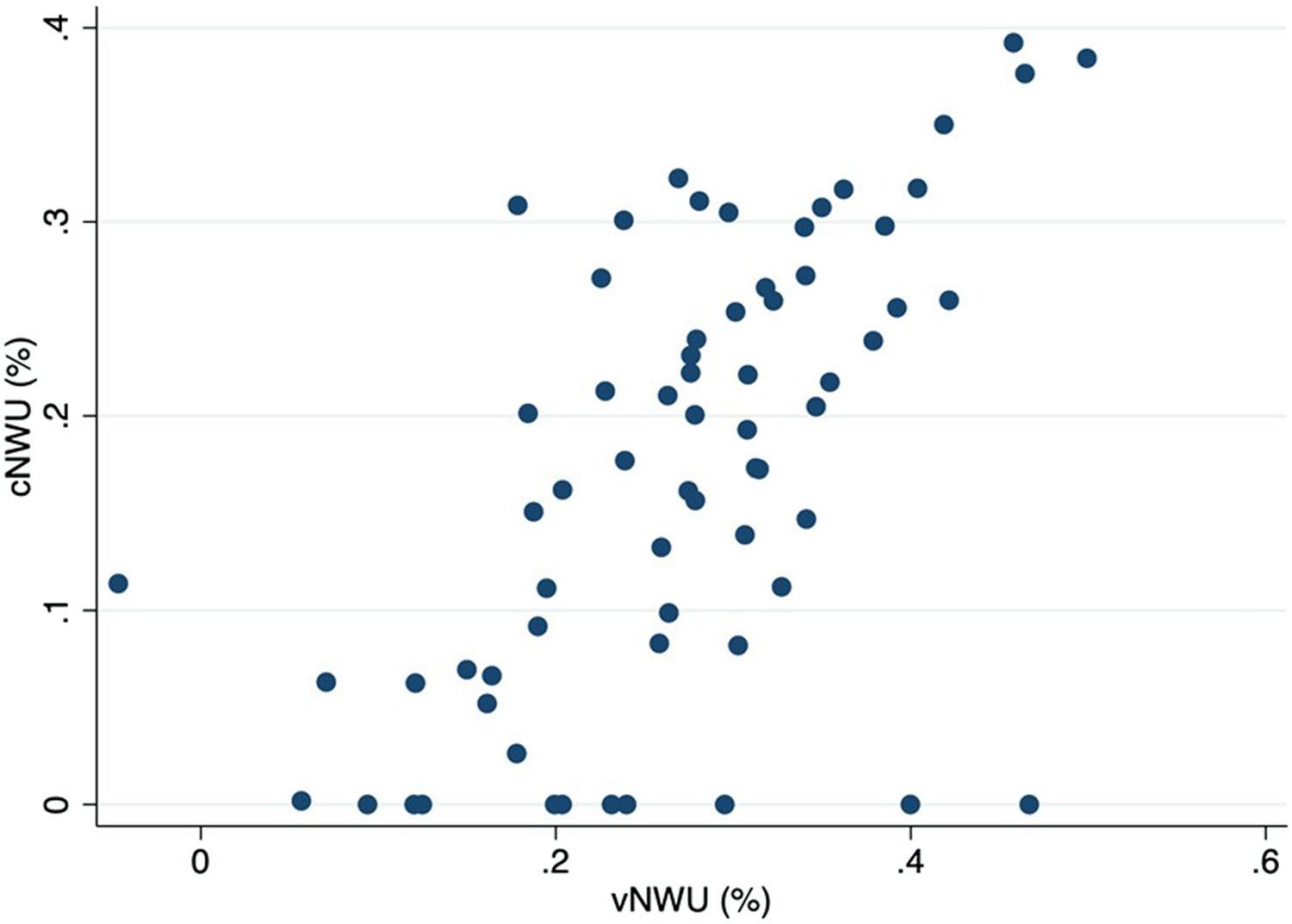
Depiction of correlation between conventional (cNWU) and virtual non-contrast net water uptake (vNWU) for edema underestimation in %. cNWU and vNWU show a strong correlation (r = 0.71, *p* < 0.0001).

The median cNWU was 19.7% (IQR: 8.2–26.6%), and the median vNWU was 27.6% (IQR: 19.9–34.0%), which was statistically not different (*p* = 0.57). The median edema underestimation, described as the difference between cNWU and vNWU was 8.7% (IQR: 4.5–14.1%, range: 1.6–46.6%). cNWU and vNWU showed a strong correlation (r = 0.71, *p* < 0.0001) (Fig. 2). In 39 (56%) patients, edema underestimation was below 10%.

### Independent variables associated with edema underestimation

**Figure 3:**
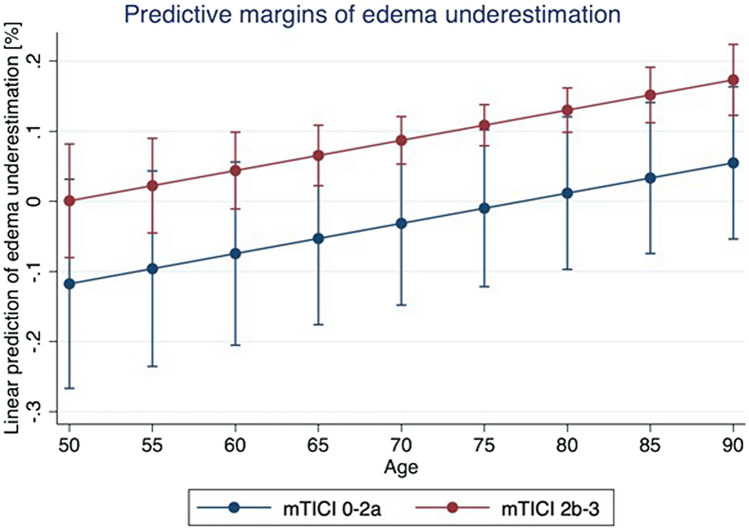
Relationship of successful recanalization and age on the degree of edema underestimation in %. Elderly patients show an increase in edema underestimation, irrespectively of successful or unsuccessful recanalization. Overall, successful recanalization was associated with higher edema underestimation. mTICI modified Thrombolysis in Cerebral Infarction.

Multivariable linear regression analysis was used to test the association between baseline and treatment variables with the difference between cNWU and vNWU as a measure of edema underestimation (Table 2). Successful recanalization (ß: 11.8%, 95% CI 0.27–23.4, *p* = 0.04), and age (ß: 0.04%, 95% CI 0.01–0.07, *p* = 0.005) were significantly associated with edema underestimation. In multivariable linear regression, time from symptom onset to flow restoration did not show significant difference between groups. The relationship between age and successful recanalization as well edema underestimation is shown in Fig. 3.

**Table 2:** Multivariable linear regression analysis to determine independent variables associated with edema underestimation.

	Beta coefficient	95% confidence interval	*p* value
Age (years)	0.43	0.14, 0.72	0.005*
Blood Glucose (mg/dl)	0.025	− 0.001, 0.13	0.64
Admission ASPECTS (per point)	0.03	− 0.01, 0.2	0.51
Successful recanalisation (≥ mTICI2b)	11.8	0.27, 23.4	0.04*
Time from symptom onset to flow restoration (min)	0.028	0.003, 0.04	0.88

n = 70 patients were included in multivariable regression analysis. Age, Blood Glucose, Admission ASPECTS and Time from symptom onset to flow restoration was calculated per 1 respective unit. Successful recanalization is defined as mTICI 2b or higher. ASPECTS Alberta Stroke Programme Early CT Score. * Indicates significant difference between groups.

### Functional outcome depending on NWU and the degree of underestimation

**Figure 4:**
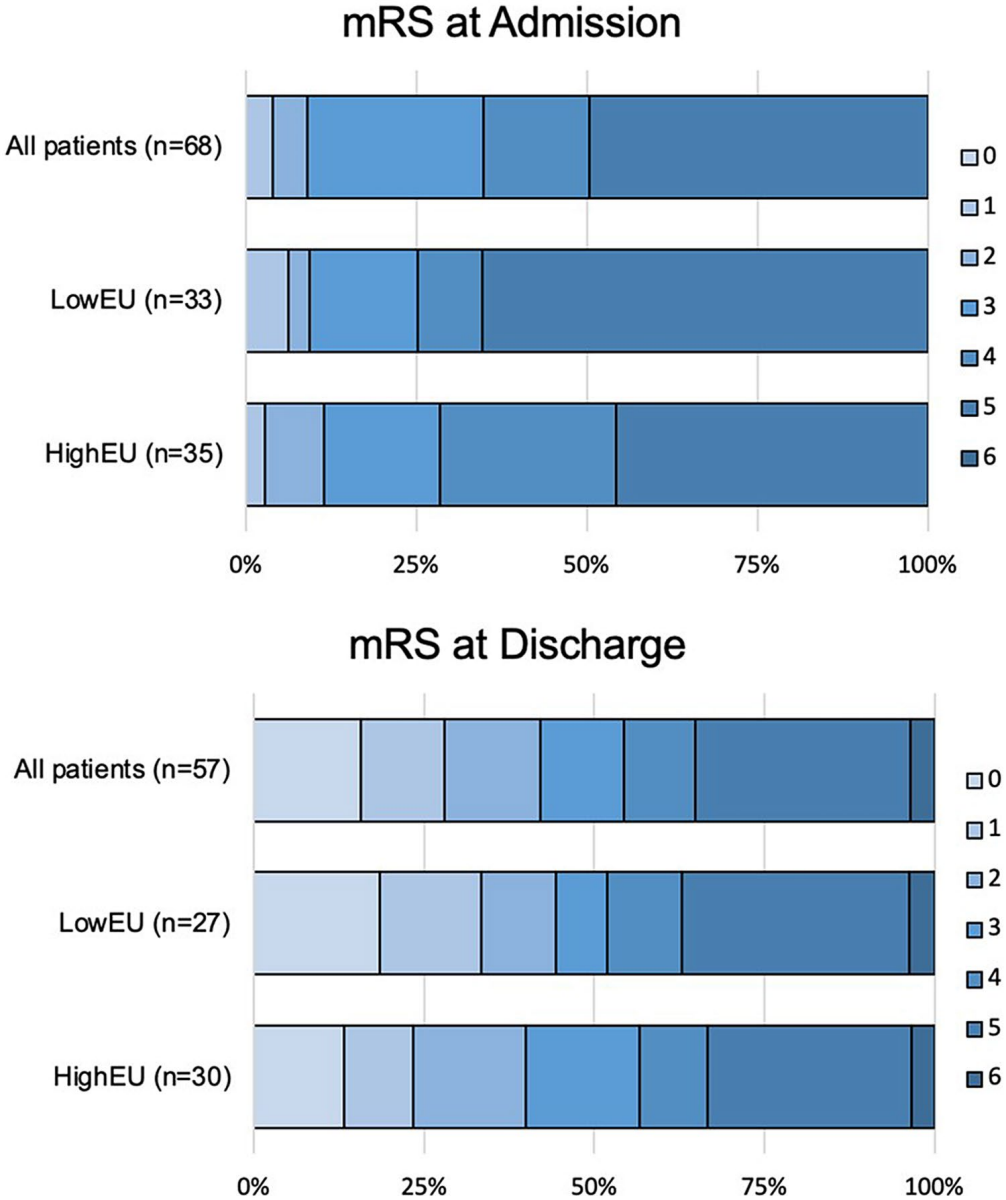
Distribution of modified Rankin Scale at admission and at discharge stratified by low (< 8.7%, LowEU) and high (≥ 8.7%, HighEU) edema underestimation. *Shown is the distribution of modified Rankin Scale (mRS) which range from 0 (no symptoms) to 6 (death*). The rate of good functional outcome (mRS 0–2 at discharge) was 40% for patients with high edema underestimation compared to 45% good outcome for patients with low edema underestimation (*p* = 0.54).

The rate of good functional outcome (mRS 0–2 at discharge) was 40% in patients with high edema underestimation compared to 45% good outcome in patients with low edema underestimation (Fig. 4). The median NIHSS at discharge was 5 in both groups (IQR 1.00–16.50 for patients with low edema underestimation and IQR 2.00–14.00 for patients with high edema underestimation), which was not significantly different *p* = 0.92. Similarly, the median mRS at discharge was also not different between groups (*p* = 0.87) (Table 1).

Comparing both multivariable logistic regression models (model 1: +cNWU; model 2: +vNWU), the AUC to distinguish good functional outcome was not different (AUC model 1: 0.78; AUC model 2: 0.77).

## Discussion

The main purpose of this study was to investigate whether the use of conventional CT scans to measure ischemic edema may result in underestimation due to the presence of contrast staining or hemorrhage. In this study, the viability of VNC-I as a superior alternative to CP-I for NWU measurements is explored to mitigate the negative effects of contrast staining on NWU and reduce the underestimation of ischemic edema. While previous research has highlighted the clinical value of NWU as an imaging biomarker^10,25,26^, it is uncertain whether VNC-I has any benefits over CP-I for quantifying ischemic edema^27,28^. A pilot study investigating the use of VNC-I demonstrated that DECT may be advantageous in 20% of patients compared to conventional CT^14^, which is consistent with another study that reported the presence of hyperattenuating lesions after MT in approximately 20% of patients on 24-h follow-up CT scans^13^. It is important to acknowledge that the occurrence of contrast staining is believed to arise from a complex of multiple factors, leading to increased blood–brain-barrier permeability in infarcted brain parenchyma^29^. While Hemorrhage remains in the extravascular space for days and should be hyperdense beyond 48 h, contrast staining is time sensitive^30^ and is likely be absorbed within 48 h^12^. Prolonged contrast staining on follow-up DECT CT 48–72 h after thrombectomy seems to be associated with poor outcome, independently of recanalization status^31^. Given that vNWU is measured using VNC-I, it is likely that the differences between cNWU and vNWU are attributable to contrast staining. For the current study, no difference in time (flow restoration to follow-up CT) was seen between groups of low and high edema underestimation.

The main findings of this study were that the median underestimation of edema (difference between cNWU and vNWU) was 8.7%, and that age and successful recanalization influenced edema underestimation. Overall, correlation between cNWU and vNWU was strong. Median cNWU was 19.7% which is consistent with previously published data stating NWU values between 17.6% for successfully recanalized patients (mTICI 2b or better) and 24.9% for persistent LVO (mTICI 0–1) on follow-up CT scans^8^.

In multivariate analysis, two parameters remained significantly associated with edema underestimation: Successful recanalization and age. Recent studies showed that, successful recanalization is associated with lower ischemic edema formation^8,32^ and decreased incidence of symptomatic intracranial hemorrhage (SICH)^33^. While SICH is easily identifiable on CP-I and should always be excluded from the chosen ROI for NWU measurements, other hyperdense lesions such as contrast staining and/or hemorrhagic transformation can be obscured on conventional CT techniques. Contrast staining tends to have a lower density than hemorrhagic transformation (HU < 50 vs. HU > 90) on immediate postprocedural CT scans^34^, making it especially difficult to differentiate from vital brain parenchyma. In the current study, successful recanalization was associated with increased edema underestimation, suggestive of reperfusion injury with contrast staining. Yet, the short-term clinical outcome was not different between low and high edema underestimation, questioning this conclusion. An extensive review addressing contrast extravasation after cerebral revascularization, concluded a lack of consensus on how to best interpret these radiographic findings with some papers describing contrast staining as a predictor for poor clinical outcome while others did not find any correlation to clinical outcome^29^.

In elderly patients, the vulnerability of the vessels is increased due to lower vessel compliance^35^, resulting in more frequent hemorrhagic transformation and contrast staining^36^. Both increase the Hounsfield Units in CP-I. Especially contrast staining leads to a stronger difference to VNC-I, and thus to an increased underestimation of edema. Moreover, the combination of age-related small vessel disease and reduction of brain volume may directly impair the accuracy of densitometric assessments due to an increased number of voxels that do not represent brain parenchyma.

In the past, it has been observed that NWU measured in follow-up CT is a predictor of outcome based on quantification on CP-I. In this study, no significant difference was seen in the association between outcome and degree of edema underestimation, further strengthening the impression that both cNWU and vNWU are reasonable imaging techniques for NWU quantification. One reason for the high correlation lies within the ROIs chosen for NWU measurements. Wherever possible, the ROI was placed so that visually apparent hemorrhage or staining was not within the area used for measurement (as depicted in Fig. 1). This is an established method and important to reduce the effect of contrast staining and hemorrhage on NWU quantification^10,26,37^.

This is a retrospective observational, single-center study with several limitations. Firstly, a relatively small number of patients were included owing to inclusion requirements such as dual-energy CT as follow-up examination, which was only recently adopted for follow-up imaging at our clinic. Moreover, the quantification of NWU using DECT imaging has not been fully established, and caution should be exercised when interpreting the results for treatment analysis. Studies have demonstrated that VNC-I and iodine overlay maps can effectively distinguish between contrast agents and blood products. However, it is important to note that VNC-I cannot be directly compared to standard non-enhanced CT scans due to differences in tissue contrast. For example, the differentiation between gray and white matter may be compromised and the impact of a secondary (non-standardized) calculation of the virtual unenhanced non-contrast image on the quantification of NWU is not yet fully understood. Secondly, both patients with good prognosis (no identifiable demarcation) and patients with poor prognosis (PH2) were excluded. This might lead to outcome biases and impair generalization of the results. Thirdly, the routine use of DECT as a follow-up in acute ischemic stroke patients could increase patient radiation exposure, raising concerns in daily practice. Therefore, its application should always be carefully considered in light of the benefits. Lastly, short-term clinical outcome was evaluated as mRS at discharge whereas mRS at 90 days is often used in literature but was not obtained for this study. However, mRS at discharge strongly predicts 90 days mRS^38^.

Besides the technical implications of improving NWU quantification, the impact on outcome prediction may be of particular relevance. In this study, NWU measurements in conventional CP-I and VNC-I were highly correlated. A median edema underestimation of 8.7% was measured for cNWU, but it had no short-term clinical implication as edema underestimation was not associated with outcome at discharge using both NIHSS and mRS scores. VNC-I may have its use in patients with low edema formation as well as in patients with diffuse hyperattenuations, which might be hard to identify on CP-I. Future research is required to investigate the association of edema underestimation with long-term functional outcome and to assess whether vNWU quantification may be used as a tool to improve the comparability of infarct imaging endpoints in ischemic stroke studies.

## Conclusion

NWU measurements in conventional follow-up CT was highly correlated to NWU measurements in DECT-based VNC-I maps. A median edema underestimation of 8.7% was observed but without short-term clinical implications. The results confirm that CP-I as well as VNC-I can be used to quantify NWU reliably. Further research is needed for long-term clinical implications.

## Data Availability

The data that support the results of this study are available from the corresponding author upon reasonable request.

## Abbreviations

ASPECTS: Alberta Stroke Programme Early CT Score
CP-I: Polychromatic computed tomography images
DECT: Dual-energy computed tomography
ESMINT: European Society of Minimally Invasive Neurological Therapy
ESO: European Stroke Organization
LVO: Large vessel occlusion
MT: Mechanical thrombectomy
mTICI: Modified thrombolysis in cerebral infarction
NIHSS: National Institutes Health Stroke Scale
NWU: Net water uptake
ROI: Region of interest
VNC-I: Virtual non-contrast images
